# Thermally insensitive determination of the linewidth broadening factor in nanostructured semiconductor lasers using optical injection locking

**DOI:** 10.1038/srep27825

**Published:** 2016-06-15

**Authors:** Cheng Wang, Kevin Schires, Marek Osiński, Philip J. Poole, Frédéric Grillot

**Affiliations:** 1 CNRS LTCI, Télécom ParisTech, Université Paris-Saclay, 46 Rue Barrault, 75634 Paris Cedex 13, France; 2 Center for High Technology Materials, University of New Mexico, 1313 Goddard St. SE, Albuquerque, NM 87106-4343, USA; 3 National Research Council of Canada, Ottawa, ON, K1A 0R6 Canada

## Abstract

In semiconductor lasers, current injection not only provides the optical gain, but also induces variation of the refractive index, as governed by the Kramers-Krönig relation. The linear coupling between the changes of the effective refractive index and the modal gain is described by the linewidth broadening factor, which is responsible for many static and dynamic features of semiconductor lasers. Intensive efforts have been made to characterize this factor in the past three decades. In this paper, we propose a simple, flexible technique for measuring the linewidth broadening factor of semiconductor lasers. It relies on the stable optical injection locking of semiconductor lasers, and the linewidth broadening factor is extracted from the residual side-modes, which are supported by the amplified spontaneous emission. This new technique has great advantages of insensitivity to thermal effects, the bias current, and the choice of injection-locked mode. In addition, it does not require the explicit knowledge of optical injection conditions, including the injection strength and the frequency detuning. The standard deviation of the measurements is less than 15%.

Quantum fluctuations associated with the lasing process affect both the intensity and the phase of the optical field. While all lasers experience phase fluctuations caused by spontaneous emission, carrier fluctuations in semiconductor lasers provide a second mechanism of phase fluctuations due to the coupling between the carrier density, the optical gain, and the refractive index in the optical cavity. This phase-amplitude coupling is a key feature distinguishing semiconductor lasers from any other type of lasers, and is responsible for a much broader linewidth in semiconductor lasers. The parameter that quantifies the magnitude of this effect is the linewidth broadening factor (LBF), also known as α_H_-factor, introduced in the context of linewidth analysis by Henry^1^. It describes the coupling between the carrier-induced variation of real and imaginary parts of the effective susceptibility χ^2^:


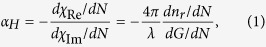


where *N* is the carrier density and λ is the lasing wavelength. The real part 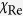 determines the effective refractive index 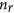, while the imaginary part 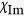 determines the modal gain *G* of the laser. In contrast to the fundamental quantum limit originally proposed by Schawlow and Townes taking into account the spontaneous emission^3^, the spectral linewidth in a semiconductor laser is broadened by a factor of (1 + 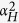)^4^. Semiconductor lasers with bulk and quantum-well (Qwell) active regions usually have LBFs of 2.0~5.0 2. Intersubband quantum cascade lasers, based on multiple-Qwell or superlattice structures, exhibit small values of LBF owing to the intersubband optical transitions and the discrete energy levels^5^,^6^. In contrast, low-dimensional nanostructured quantum dash (Qdash) and quantum-dot (Qdot) lasers have much more complex carrier dynamics^7^, leading to a wide spread of LBF values from near-zero up to more than 10^8^,^9^.

Extensive studies have shown that the LBF also affects many other features of semiconductor lasers, such as the frequency chirping under current modulation^2^, mode stability against optical feedback^10^, nonlinear dynamics under optical injection^11^, four-wave mixing generation^12^, as well as the filamentation in broad-area semiconductor lasers^13^. Many efforts have been made for the characterization of the LBF. Osiński and Buus^14^ and Villafranca *et al*.^15^ overviewed various measurement techniques. These techniques can be classified into three types: (I) Analysis of the optical spectrum, including the *optical linewidth* method^1^ and the *Hakki-Paoli* method^16^, relying on simultaneous observation of the longitudinal mode wavelength shift and the modal gain changes with varying current below lasing threshold. (II) High-frequency modulation, including the *FM*/*AM* method^17^,^18^ and the *Fiber Transfer Function* method^19^. (III) External optical control, including the *optical injection*^20^, the *optical feedback*^21^, and the *four-wave mixing* methods^22^. Although all the above-threshold techniques can be used under the exactly controlled operating conditions of the device, the extracted LBF results from a complex combination of multiple effects. For instance, above the lasing threshold, the LBF becomes an effective parameter dependent on the optical power^23^,^24^, the type of cavity (distributed feedback vs Fabry-Perot)^25^,^26^ and the gain nonlinearities due to spectral hole burning, spatial hole burning, and carrier-heating effects^27^,^28^,^29^. Consequently, the LBF measured above threshold may mask the basic features of the semiconductor material. Therefore, it is still of interest to access asymptotic values of the LBF parameter close to threshold, in particular to probe its dependence on carrier concentration and photon energy that cannot be varied in the real operating conditions above threshold.

To start with, we briefly review the Hakki-Paoli method and the optical injection method, which is helpful for the understanding of the novel method proposed in this paper. The Hakki-Paoli method relies on the recording of sub-threshold amplified spontaneous emission spectrum. Through measuring the changes of the wavelength shift and the modal gain of the Fabry-Perot (FP) modes with the current, the LBF defined in Eq. (1) is equivalently determined by^30^


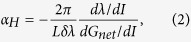


where *L* is the cavity length, δλ is the mode spacing, and *d*λ and *dG*_*net*_ are respectively the mode wavelength and net modal gain variations caused by the current change *dI*. Figure 1a presents a set of sub-threshold gain spectra, obtained from a *multipopulation rate equation* model^31^,^32^. It shows that when the pump current decreases from the lasing threshold, the net modal gain *G*_*net*_ is reduced gradually from its threshold value (dashed curve). This method is relatively straightforward, but severely suffers from thermal effects, which cause a red-shift of the optical wavelength and thereby interfere with the tracking of the carrier-induced blue-shift effect. In order to reduce the thermal effects, a current source operated in the pulsed mode instead of the continuous-wave mode can be utilized, but this results in a low signal-to-noise ratio and even irregular spectral line shape, which can hinder the extraction of the LBF. On the other hand, the optical injection method utilizes the optical injection effects to extract the LBF of the injection-locked mode. When a free-running FP semiconductor laser is biased above the threshold, many cavity modes can lase simultaneously, as shown in Fig. 1b (gray lines). However, once the laser is stably locked to an external single-mode laser (master laser), only the mode subject to the optical injection keeps lasing, while all side-modes are suppressed (blue lines). The stable-locking range is bounded by Hopf bifurcation and the saddle-node bifurcation, as illustrated in Fig. 1c (green area)^33^, where the asymmetry is induced by the non-zero LBF^11^. Therefore, the LBF of the mode subject to optical injection could in principle be determined from the slope of the two bifurcations^20^. However, this measurement requires the knowledge of accurate values of the frequency detuning 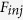 (optical frequency difference between the master and slave lasers) and the injection ratio 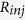 (ratio of the injected signal power 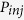 to the free-running slave laser power 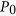, both inside the slave laser cavity), which is very challenging to implement in practice. This is because there is a mode profile mismatch between the master beam projected onto the slave laser facet and the slave guided mode, which can substantially reduce the effective injection strength.

In this paper, we present a simple, flexible method for measuring the LBF of semiconductor lasers, based on the analysis of residual side-modes of a laser under optical injection. In contrast to the aforementioned techniques, the novel proposed method does not require any sophisticated high-frequency equipment and is insensitive to thermal effects, injection conditions, or the pump current level. The measured LBF values are compared with those obtained by the Hakki-Paoli method, and are shown to have a standard deviation of less than 15%.

## Results and Discussion

**Figure 1:**
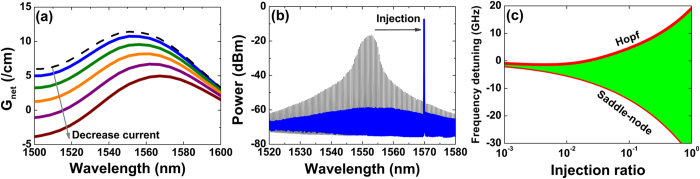
(**a**) Simulated sub-threshold net modal gain. The dashed curve is the gain at the threshold. (**b**) Measured optical spectra. The gray lines are for the free-running laser, and the blue lines are for the injection-locked laser. (**c**) Simulated stable injection-locking diagram (after ref. 33).

**Figure 2:**
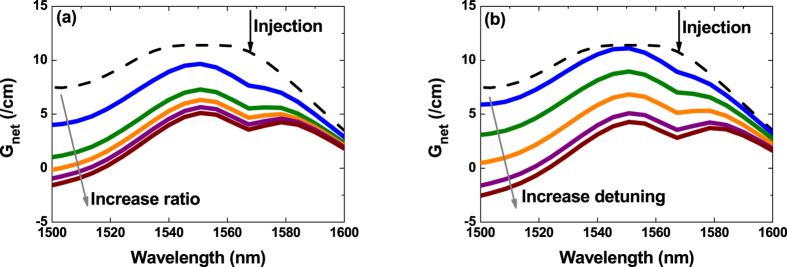
Effects of optical injection on the net modal gain spectrum of a Qdot laser. (**a**) The injection ratio R_inj_ increases from 0.3 to 2.7 by a step of +0.6, with a fixed zero wavelength detuning; (**b**) The wavelength is detuned from −99 pm to + 33 pm by a step of +33 pm with a fixed injection ratio of 2.7. The dashed lines denote the gain without optical injection.

As discussed in Fig. 1b, optical injection suppresses the side-modes in a FP semiconductor laser. In order to understand the underlying physical mechanism, Fig. 2 simulates the effects of optical injection on the net modal gain of a Qdot laser using the multipopulation rate equation model. It shows that both an enhancement of the injection ratio (Fig. 2a) and an increase in the wavelength detuning (Fig. 2b) reduce the whole net modal gain compared to that of the free-running laser (dashed curve). Consequently, in both cases the reduced gain leads to a suppression of side modes. The gain reduction effect of optical injection can be understood by examining the steady-state solutions of the classical rate equation model for injection-locked semiconductor lasers^34^


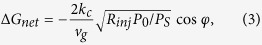


with the phase 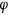 of the laser field expressed as


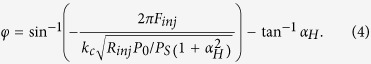


Here, 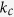 is the coupling coefficient of the master laser to the slave laser, 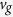 is the light group velocity, and 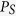 is the intra-cavity power of the slave laser under optical injection. The above equations point out that the injection ratio 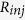 directly reduces the net modal gain *G*_*net*_ through the carrier population, while the frequency detuning 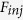 does the same indirectly through the phase of the laser field. From the physics viewpoint, optical injection pushes the cavity resonance to the longer wavelength side. Therefore, an increase in the wavelength detuning shifts the lasing mode towards the cavity resonance, and hence reduces the gain to a lower level. In addition, Fig. 2 also demonstrates that the wavelength detuning changes the net modal gain more linearly than the injection ratio, and the gain reduction behavior is similar to that induced by the pump current in the Hakki-Paoli method in (cf. Fig. 1).

From the knowledge of Kramers-Krönig relationships, the wavelength detuning of optical injection in Fig. 2b not only changes the gain of the side-modes, but also shifts the wavelengths. Consequently, we can recast the LBF expression in Eq. (2) as


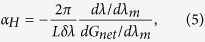


with λ_m_ being the injection wavelength of the master laser. Equation (5) provides the basis for experimental determination of the LBF using the optical injection-locking technique. A similar formula can be deduced by varying the injection power from the master laser based on Fig. 2(a). However, this approach is less flexible due to a nonlinear dependence of the gain on the injection ratio, as well as the usual power limitation of tunable laser sources. It should be noted that a method utilizing an external light to measure the LBF of a below-threshold semiconductor laser has been reported^35^, but that method takes advantage of the cavity resonance effect, which is different from the injection-locking mechanism discussed in this work.

The experiments were performed on two types of diode lasers with nanostructured active regions, incorporating either Qdashes or Qdots. Owing to a more complex electronic structure, variations of the LBF in such lasers are more pronounced, which constitutes a better configuration for the validation of the novel method proposed in this paper. The experimental setup and the nanostructured semiconductor lasers are described in the Methods section.

**Figure 3:**
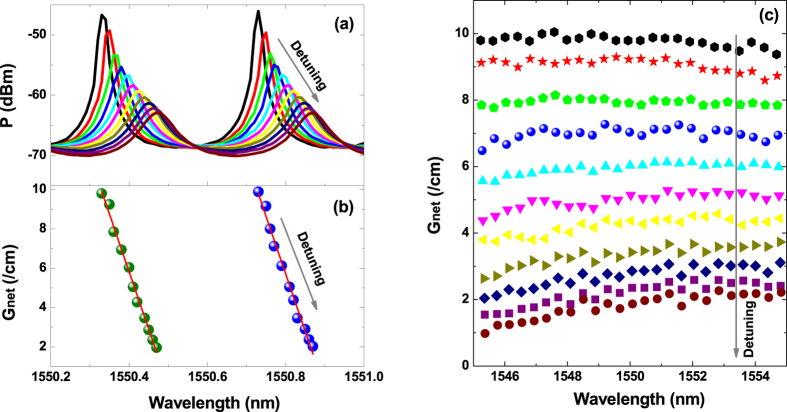
Illustration of the proposed LBF measurement technique for the Qdash laser. (**a**) Wavelength and power variations of two side-modes with detuning of the injected light from shorter to longer wavelength within the stable-locking range. (**b**) The corresponding net modal gain (Gnet) as a function of the mode peak wavelength (λ). The red lines indicate the least square linear fitting. (**c**) Measured Gnet versus λ over a span of 1545–1555 nm.

We first illustrate the proposed technique taking an example of the Qdash laser described in the Methods section. The laser had a lasing threshold of *I*_th_ = 45 mA, and the gain peak was located around 1550 nm. Figure 3 shows the LBF measurement procedure for the Qdash laser biased at 60 mA. The optical injection with a fixed incident power of 6.1 mW was applied to a longitudinal mode at about 20 nm away (~1570 nm) from the gain peak. When the master laser was detuned from a shorter wavelength (Hopf point side) to a longer wavelength (saddle-node point side) within the stable-locking range, the side-modes varied correspondingly as shown in Fig. 3a. The magnitude of the side-modes decreased, indicating the gain was reduced. At the same time, the wavelength was red-shifted due to an increase of the refractive index, since the optical injection reduces the carrier density. The net modal gain *G*_net_ was extracted from the power ratios of adjacent peaks and valleys of the optical spectrum, as in the Hakki-Paoli method^30^. Figure 3b shows the extracted *G*_*net*_ for each side-mode in Fig. 3a as a function of the wavelength λ. It can be seen that *G*_*net*_ was reduced almost linearly from 10 cm^−1^ down to 2 cm^−1^ with the red-shift of the wavelength. Linear fitting (red line) provides the ratio of *d*λ/*dG*_*net*_, which is used for the calculation of the LBF through Eq. (5). Figure 3c presents the net modal gain spectra of the Qdash laser within the span of 1545–1555 nm corresponding to the window for the LBF extraction. With the increase in the wavelength detuning, the whole gain spectrum falls down gradually, as in the simulation result of Fig. 2b. In addition, the gain reduces faster at shorter wavelengths than at longer ones. For the same injection conditions, *G*_*net*_ at 1545.3 nm decreases from 9.8 cm^−1^ down to 1.0 cm^−1^, while *G*_*net*_ at 1554.8 nm decreases from 9.4 cm^−1^ down to 2.2 cm^−1^. The asymmetry of the gain spectra implies that the laser has a larger differential gain (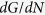) at the shorter wavelength side.

**Figure 4:**
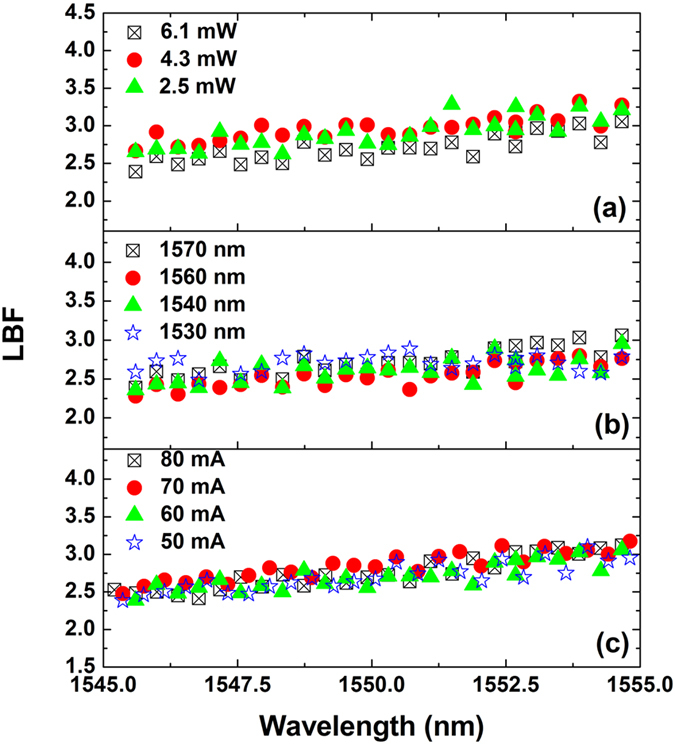
Measured LBF of the Qdash laser under various conditions. (**a**) Varying optical injection power. The injected mode was around 1570 nm, and the laser was biased at 60 mA. (**b**) Different injected modes. The injection power was 6.1 mW, and the laser was biased at 60 mA. (**c**) Varying bias current. The injected mode was around 1570 nm, and the injection power was 6.1 mW. The standard deviations of the measurements in (**a**–**c**) are 12%, 11%, and 9%, respectively.

Figure 4 shows the measured LBF of the Qdash laser under different operation conditions. Generally, the LBF increases from about 2.5 at 1545 nm to about 3.0 at 1555 nm. Figure 4a demonstrates that the measured LBF values do not change much for different optical injection powers. The standard deviation of the measured LBFs at three injection powers of 6.1, 4.3, and 2.5 mW was about 12%. Figure 4b reveals that the measurement is not sensitive to the choice of the injected mode. The standard deviation of the LBFs at four injected modes (at 1570, 1560, 1540, and 1530 nm) was about 11%. In addition, the method is also insensitive to the bias current, as illustrated in Fig. 4c, and the standard deviation of the LBFs at four bias currents (80, 70, 60, 50 mA) was about 9%. The insensitivity to the above conditions can be attributed to the fact that the side-modes under measurement are always non-lasing, even though the injected mode is lasing, meaning that the side-modes are operated below-threshold. Therefore, this technique can be a superior complement of the sub-threshold Hakki-Paoli method, with one important advantage. The Hakki-Paoli method relies on the change of the bias current, which always results in device heating and thus complicates the accurate extraction of the LBF. In contrast, the method proposed in this paper utilizes a fixed pump current with a constant associated heating, and hence remains insensitive to thermal effects.

**Figure 5:**
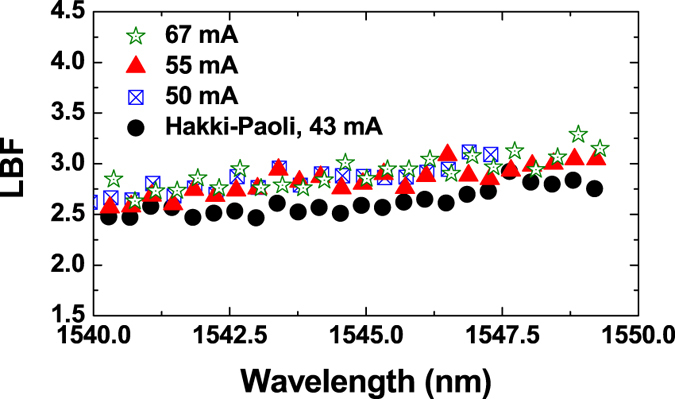
Measured LBF of the Qdot laser at different currents. The injected mode was around 1570 nm, and the injection power was 8.0 mW. The black circles represent the LBF measured by the Hakki-Paoli method near the lasing threshold at 43 mA.

The LBF measurements using the proposed method are compared with the Hakki-Paoli method in Fig. 5 for a Qdot laser described in the Methods section. The Qdot laser’s lasing threshold was 43 mA, with a gain peak around 1545 nm. In the Hakki-Paoli measurements, the pump current source was operated in the continuous-wave mode, and the sub-threshold heating was evaluated from that above threshold^36^. The LBF measured under side-mode injection locking conditions at three bias currents (67, 55, and 50 mA) increases from about 2.7 at 1540 nm to about 3.2 at 1549 nm. As shown in Fig. 5, the LBF measured by the novel method is in a relatively good agreement with that obtained by the Hakki-Paoli method (black circles), albeit it is slightly higher. This can be attributed to the underestimation of the thermal effects in the latter method^36^. It is noted that the LBFs of the Qdot laser are similar to those of the Qdash laser shown in Fig. 4, although carriers in the Qdots are confined in all three dimensions. This can be attributed to the inhomogeneous broadening effects, originating from the large dot size dispersion^37^.

In conclusion, we have proposed a novel method for measuring the sub-threshold LBF of semiconductor lasers. It relies on the analysis of the residual side-modes of the laser subject to an optical injection. The method is insensitive to thermal effects, bias current, and injection conditions. The standard deviation of the measurements does not exceed 15%, and the measured values are in good agreement with those obtained using the standard Hakki-Paoli method. The proposed technique may be of prime importance for advanced types of laser devices, such as quantum cascade lasers^38^,^39^,^40^ and lasers on silicon^41^,^42^, for which the LBF behavior does require a better understanding and vivid discussions dealing with the thermal effects still persist.

## Methods

**Figure 6:**
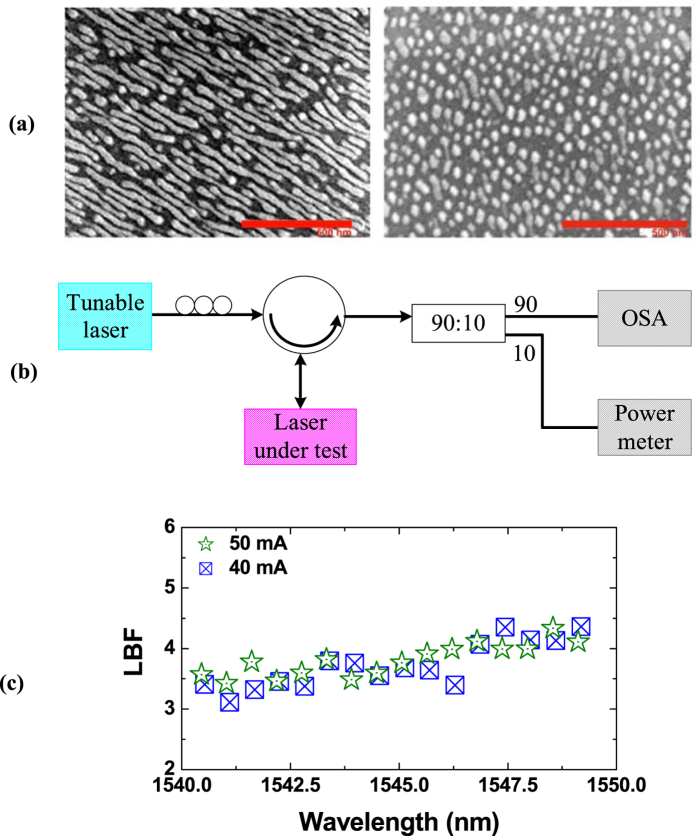
(**a**) SEM images of the InAs Qdash (left) and Qdot (right) nanostructures (scale bar is 500 nm). (**b**) Experimental setup for measuring the LBF. (**c**) Measured LBF of a commercial Qwell laser. The lasing threshold is 24 mA, and the lasing spectrum peaks around 1545 nm.

Figure 6a shows the scanning electron microscope (SEM) images of the InAs Qdashes (left) and Qdots (right), grown by chemical beam epitaxy (CBE) on InP substrates^43^. A thin layer of GaAs (GaP) underneath InAs was used to promote the Qdash (Qdot) morphology. The dashes were typically elongated nanostructures of a few hundreds of nanometers in length, while the dots were more symmetric in shape, with a diameter on the order of 40 nm^44^. The laser structures were grown on (001)-oriented *n*-type InP substrates. An *n*-type cladding was first grown, followed by a 350-nm-thick 1.15Q waveguiding core containing 5 layers of dashes/dots. An upper *p*-type InP cladding layer was then grown (containing an etch stop for ridge fabrication), followed by a heavily doped *p*-type InGaAs contact layer. Single-mode ridge waveguide lasers were then fabricated, cleaved, and mounted for testing. Both lasers had a cavity length of 1 mm, with the Qdash FP laser having a ridge width of 2 μm, and the Qdot FP laser having a ridge width of 3 μm.

Figure 6b illustrates the experimental setup for measuring the LBF. A tunable external cavity laser (Anritsu FP001168) was used as the master laser, and the injected light was coupled into the laser under test (slave laser) through an optical circulator. The polarizations of both lasers were aligned by a polarization controller. The optical spectrum of the output light (90%) was captured by a high resolution (10 pm) optical spectrum analyzer (OSA), and 10% of the output power was monitored by a power meter. The temperature of the slave laser was maintained at 20 °C throughout the experiment. Figure 6c presents the measured LBF of a commercial Qwell FP laser (Alcatel-Lucent) at two bias currents (40 and 50 mA) using the experimental setup. The threshold current of the laser was 24 mA, and the peak of the optical spectrum was around 1545 nm. Within the spectral range of 1540–1550 nm, the LBFs of the Qwell laser vary between 3.1 and 4.4, which are roughly 30% larger than those of the Qdash and Qdot lasers in Figs 4 and 5.

## Additional Information

**How to cite this article**: Wang, C. *et al*. Thermally insensitive determination of the linewidth broadening factor in nanostructured semiconductor lasers using optical injection locking. *Sci. Rep.* **6**, 27825; doi: 10.1038/srep27825 (2016).
